# Iohexol as an Emerging Contaminant Potentially Influencing Structural and Functional Changes in the Activated Sludge Microbiome

**DOI:** 10.3390/molecules30234641

**Published:** 2025-12-03

**Authors:** Agnieszka Nowak, Magdalena Pacwa-Płociniczak, Urszula Guzik, Danuta Wojcieszyńska

**Affiliations:** Institute of Biology, Biotechnology and Environmental Protection, Faculty of Natural Science, University of Silesia in Katowice, Jagiellońska 28, 40-032 Katowice, Poland; magdalena.pacwa-plociniczak@us.edu.pl (M.P.-P.); urszula.guzik@us.edu.pl (U.G.); danuta.wojcieszynska@us.edu.pl (D.W.)

**Keywords:** iohexol, activated sludge, microbial community, biotransformation, functional diversity

## Abstract

Iohexol (IOX) is one of the most commonly used iodinated contrast agents due to its relatively low toxicity. However, owing to its low biodegradability, it accumulates in wastewater and sewage sludge, ultimately reaching wastewater treatment plants. With increasing IOX loads, the activated sludge (AS) microbiome is exposed to its long-term effects. Therefore, this study aimed to determine the impact of IOX on the structure and functioning of AS. The results demonstrated that IOX at lower concentrations does not affect the functional parameters of AS, and only long-term exposure of the microbiome to a concentration of 20 mg/L results in functional changes. These changes themselves, among other effects, concerning amino acid and carbohydrate metabolism and the phosphorus cycle. Furthermore, the study demonstrated that despite the activated sludge microbiome’s ability to transform 20 mg/L of IOX, long-term exposure to this concentration reduces the efficiency of this process. This indicates that although IOX is a relatively safe compound, prolonged exposure of the AS microbiome to high doses may lead to functional changes in AS and potentially impair wastewater treatment processes.

## 1. Introduction

Contrast agents are substances that temporarily improve the visibility of internal body structures during imaging studies such as computed tomography (CT) or magnetic resonance imaging (MRI). Currently, over 400 million such examinations are performed worldwide annually [1,2].

Magnetic resonance imaging (MRI) contrast agents are classified based on their magnetic properties, chemical composition, including the presence or absence of metal atoms, route of administration, biodistribution, and their effect on the MRI image. Chemically, MRI contrast agents can be divided into two classes. The first class includes paramagnetic compounds, including lanthanides such as gadolinium (III). The second group includes agents containing transition elements such as manganese and iron. However, it has been observed that lanthanide salts (including gadolinium) typically hydrolyze to form hydroxides, which are taken up by the reticuloendothelial system (RES) and accumulate in the body, particularly in the liver, spleen, and bones, potentially causing toxicity. To reduce the toxicity of such contrast agents, their less toxic form, chelated complexes, is primarily used. There are three types of such complexes: non-ionic and hydrophilic complexes, ionic and hydrophilic complexes, and ionic and lipophilic complexes [3].

Most contrast agents currently used in computed tomography (CT) are chemical modifications of the 2,4,6-triiodinated benzene ring. They are classified based on their physical and chemical properties, including chemical structure, osmolality, iodine content, and ionization in solution. The toxicity of triiodobenzoic acid is reduced by introducing side chains at the 3- and 5-positions. Iodinated agents are divided into high-osmolarity ionic agents and low-osmolarity non-ionic agents based on the presence of substituents. Ionic agents typically have a well-documented risk of adverse events, some of which can be life-threatening. Non-ionic agents, on the other hand, have a significantly lower rate of serious complications [4].

Among widely used contrast agents, approximately one-quarter are iodine-based compounds, such as iohexol or diatrizoate [1,2]. These compounds must be administered in large doses during diagnostic tests, sometimes reaching up to 200 g per session. Iodinated contrast media (ICMs) are not metabolised in the body and are completely excreted in the urine after 24 h [5]. Consequently, they are present in sewage. For example, the concentration of iomeprol in hospital wastewater has been reported to range from 424 to over 2000 μg/L [6]. Currently, the development of analytical methods enables the rapid and sensitive detection of these compounds in water, resulting in their increasingly frequent identification in wastewater and surface waters. The development of a rapid and sensitive direct injection liquid chromatography (LC-MS/MS) method enables the simultaneous analysis of popular ICMs such as iopamidol, ioxitalamic acid, diatrizoic acid, iothalamic acid, iohexol, iomeprol, and iopromide in complex aqueous matrices. This method is also suitable for tracing intermediates formed during chemical oxidation of ICMs [7].

Due to their chemical structure, ICMs are highly stable in the environment. ICMs are derivatives of 2,4,6-triiodobenzoic acid containing carboxyl and hydroxyl substituents on their side chains and three iodine atoms attached to the aromatic ring [8]. This structure prevents their effective removal in conventional wastewater treatment plants due to their polarity and resistance to biological degradation. In the outflows of sewage treatment plants in Germany, the concentration of iomeprol has been reported at 13 μg/L, contributing to the presence of ICMs in surface waters and even drinking water [9]. Iodine contrast agents have been monitored in the Rhine River for 20 years. In 2020, the daily load of ICMs in the Lobith area ranged between 10.3 and 175 kg/day, with the average load of IOX estimated at almost 40 kg/day [10].

Currently, new technologies are being developed to remove contrast agents from wastewater. Unfortunately, biological methods are ineffective. For example, in the transformation of IOX, only the oxidation of alcohol groups, cleavage of the carbon–nitrogen bond, decarboxylation, oxidation of the methyl group, and cleavage of propanoic and acetic acid chains occur [8].

The use of physicochemical processes to purify wastewater from contrast agents is generating considerable interest. For example, Zinicovscaia et al. [11] used adsorption on yeast biomass and titanosilicate, achieving maximum gadolinium removal after applying extremely low pH. Furthermore, the use of Manganese Ferrite Nanoparticles allowed for over 90% gadolinium removal within 1 h in both fresh and saltwater [12]. In turn, the use of advanced oxidation methods allows for the removal of side chains and deiodination; however, no cleavage of the aromatic ring is noted [8,9]. The future may lie in the acquisition of biogenic nanoparticles and their use in contrast agent deiodination processes. It has been demonstrated, among other things, that biopalladium nanoparticles can electroreduce diatrizoate, leading to its complete deiodination. It is suggested that the catalytic deiodination of ICM, due to its efficiency and selectivity, may be an alternative to previously used chemical purification methods [8].

The presence of ICMs and their intermediates in the environment may affect living organisms. Although these compounds are not reported to cause adverse effects after short-term exposure, their chronic impact remains poorly understood [2,13]. However, Singh et al. [14] indicate that, due to structural similarities between ICMs and endocrine compounds, iodine contrast media may interact with thyroid hormone receptors, potentially disrupting the hormonal balance in fish [14]. Moreover, it has been demonstrated that, although the parent ICMs exhibit low toxicity, the products formed during photochemical degradation of ICMs may have mutagenic and genotoxic effects [2,13].

Due to the potential effects of ICMs, it is essential to assess the impact of these compounds on the AS microbiome, which is responsible for their transformation in sewage treatment plants. Therefore, this study aimed to determine the effects of IOX, a commonly used ICM, on the structure and functioning of the AS microbiome. For this purpose, changes in the metabolic activity of AS and the activity of dehydrogenases, which play a key role in the degradation of xenobiotics, were used as indicators of functional alterations. Simultaneously, the transformation of IOX during the experiment was monitored. Changes in the transcripts of the *16S rRNA*, *CYP153*, and *C23O* genes were assumed to be indicators of structural changes. These studies provide new insights into the potential hazards associated with high ICM loads in sewage.

## 2. Results and Discussion

The exceptional resistance of iodine contrast agents to degradation contributes to their increasing presence in sewage treatment plant effluents and natural environments. Although these compounds are considered to have low toxicity due to their stable structure, it is already known that they can undergo transformation during processes such as water disinfection, which leads to increased toxicity. Consequently, growing attention is being paid to their impact on the AS microbiome, which is exposed both to their effects and the processes of their biotransformation [1,15].

In this study, AS was exposed to three concentrations of IOX (0.2, 2, and 20 mg/L) over an extended period of 100 days, and its effects on the functional and structural characteristics of the AS were analysed. In the first phase of the study, the biotransformation capacity of AS toward IOX was investigated. Literature data indicate that cooperation between bacteria and AS-associated fungi enhances the biotransformation of contrast agents. Extracellular lignolytic enzymes are likely responsible for the initial transformation of the aromatic ring, while further stages of degradation are carried out by bacteria [2].

**Figure 1:**
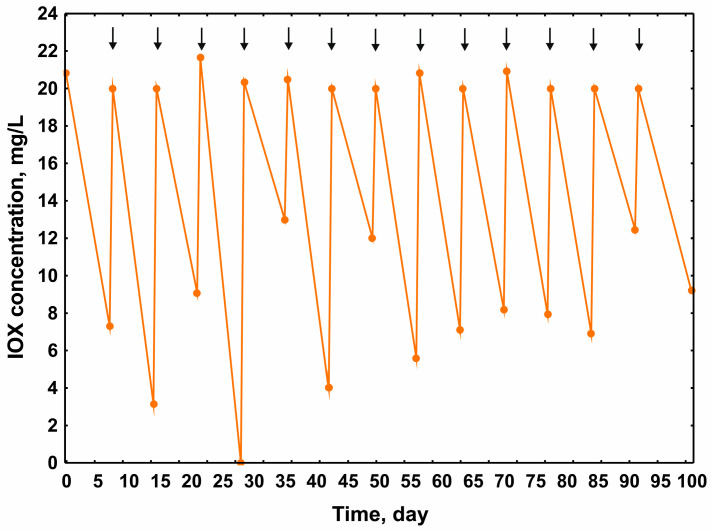
Changes in IOX concentration in AS during a 100-day experiment. IOX, iohexol; AS, activated sludge. Arrows indicate the introduction of the next doses of IOX. LOD/LOQ = 3.03.

It was observed that, initially, each administered dose of 20 mg/L IOX was efficiently biotransformed, achieving complete transformation (100%) by the 28th day of the experiment (4th dose). However, after this period, the efficiency of IOX transformation by AS decreased, reaching approximately 50% by the 100th day (Figure 1).

These results may suggest that exposure to IOX may induce changes within the AS microbiome that affect its functioning. Accordingly, a range of functional parameters of the AS were analysed in subsequent stages. The results of the Shannon diversity analysis were unexpected, as they indicated that despite an initial decline in biodiversity, a significant increase was observed on day 100 in the system treated with 20 mg/L IOX (Table 1).

This effect may be attributed to the establishment of an equilibrium within the AS ecosystem and a reduction in the abundance of dominant physiological groups. Such a shift likely facilitated the detection of activity from less prevalent microbial populations, making them more detectable, and contributed to an overall increase in functional diversity. Nevertheless, changes in the functional diversity of the AS microbiome were probably not directly related to IOX degradation.

Analysis of the sewage after the completion of the 14th and last cycle showed that the SVI, an indicator of sedimentation capacity and structural stability of the sludge, ranged between 70 and 100 mL/g (Table 2), indicating a normally functioning AS. Only in the system treated with 0.2 mg/L IOX did this index decrease to 68 mL/g, suggesting a slight deformation of the AS, potentially involving the formation of point flocs [16].

COD and BOD analysis indicated a reduction in these indicators. After the last cycle of the experiment, BOD decreased by 80% in the system with 2 mg/L IOX and by 60% in the system with 20 mg/L IOX. Similarly, COD decreased by 55% and 48%, respectively, indicating the occurrence of both biodegradation processes and chemical oxidation in the system [17]. This is further confirmed by the TOC reduction. Additionally, analysis of nitrogen compound concentrations indicated that nitrification processes were not disturbed (Table 2). Based on these analyses, it can be concluded that IOX did not cause functional disruptions in AS, resulting in the proper functioning of biological wastewater treatment processes. Maintaining proper parameters of wastewater treatment with low degradability of IOX may indicate that this compound does not exhibit toxicity toward the AS microbiome. This is confirmed by the literature, which indicates that iodinated contrast agents may not be toxic to microorganisms [18].

Changes in AS activity were further investigated by determining the transcription levels of *16S rRNA*, *CYP153*, and *C23O* [19]. *16S rRNA* gene transcripts were used as a structural marker because rRNA abundance reflects the potential number of metabolically active bacterial cells and generally correlates with cellular biomass and ribosome content. Ribosomal RNA has long been used to identify active or growing microorganisms, as its transcription increases in proportion to metabolic activity. The relationship between 16S rRNA content and growth rate is not always linear, and some microbial activities are not strictly growth-dependent (e.g., motility, osmoregulation, oxidative stress responses, exopolysaccharide production, or conjugation). Despite these limitations, using 16S rRNA transcripts as an indicator of bacterial presence and activity in environmental samples is well supported in the literature [20,21] and remains a suitable normalizer for assessing total bacterial activity in the examined system.

**Figure 2:**
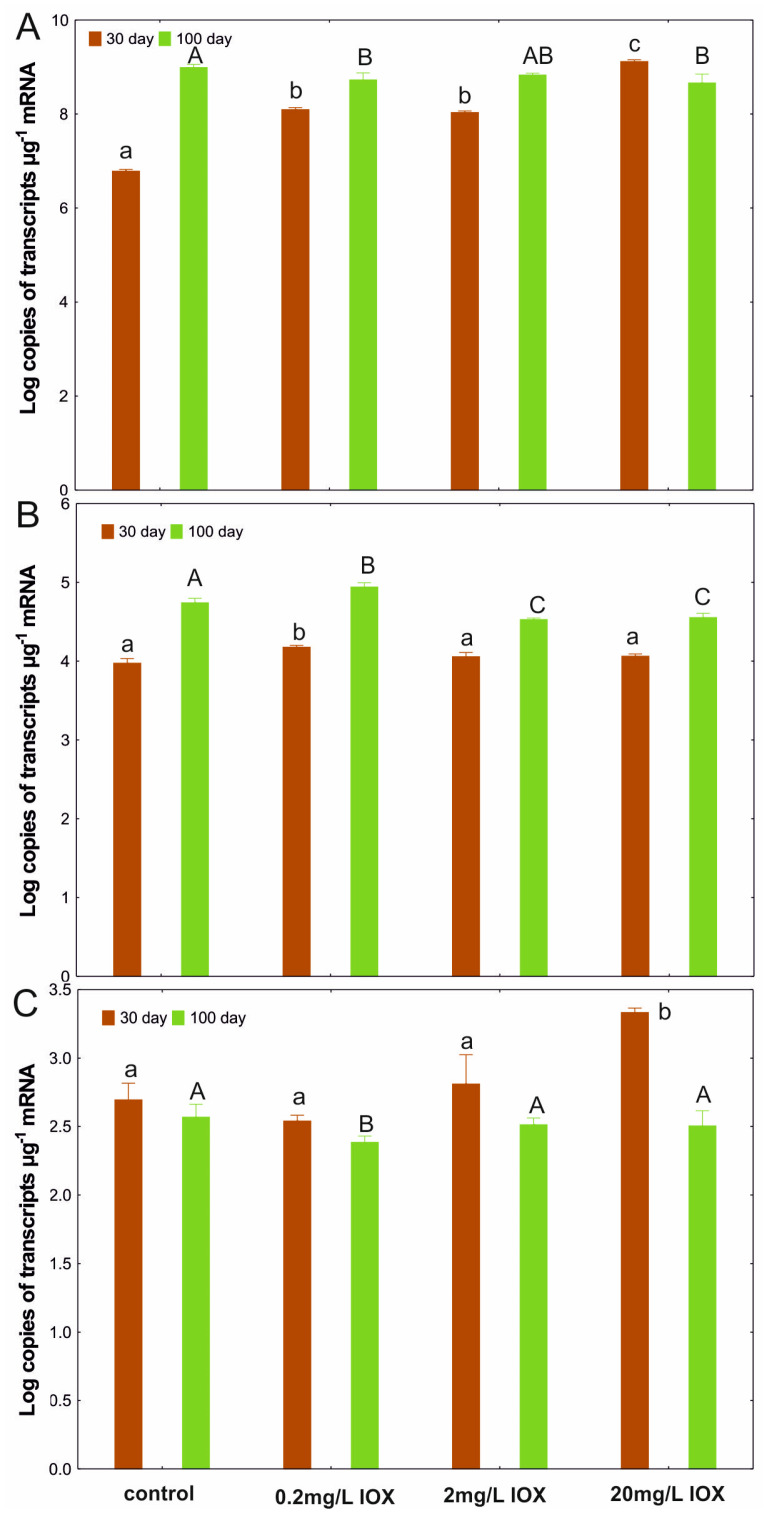
Number of *16S rRNA* (**A**), *CYP153* (**B**), and *C23O* (**C**) transcript copies in control AS, AS treated with 0.2, 2.0, or 20 mg/L IOX on days 30 and 100. Means with different letters on each sampling day are significantly different (*p* < 0.05, LSD test) considering the treatment. IOX, iohexol; AS, activated sludge; LSD, least significant difference.

A significantly increased level of *16S rRNA* gene transcription was observed in the system treated with 20 mg/L IOX after 30 days. However, by day 100, the transcription level of this gene was lower than that in the control. Similarly, a lower number of *CYP153* gene transcripts was observed in the system with 20 mg/L IOX on day 100 (Figure 2).

This may suggest a decrease in the active AS population, which would also explain the reduction in the efficiency of IOX biotransformation during the final degradation cycle. Catechol 2,3-dioxygenase is an enzyme induced by compounds with an aromatic structure and is also responsible for the cleavage of the aromatic ring [22]. It is likely that aromatic products may be formed during IOX degradation. If this hypothesis is correct, they may induce the transcription of genes involved in the corresponding degradation pathway [23]. On day 30, when the IOX transformation was at its highest (Figure 2), transcription levels of the *C23O* gene were also maximal. However, by day 100, these levels had decreased significantly (Figure 2), reflecting the weaker degradation of IOX observed during the final cycle (Figure 1).

**Figure 3:**
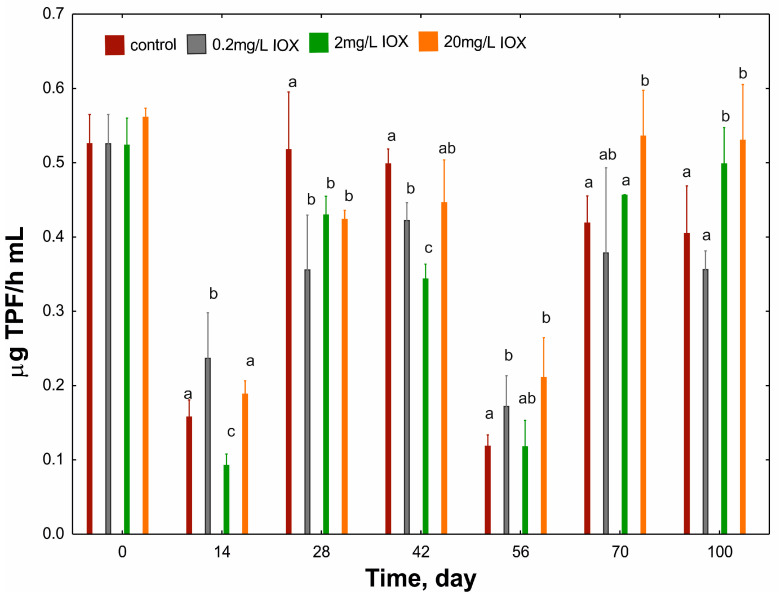
Changes in the TPF concentration (µg/mL·h) in the AS exposed to different concentrations of IOX for 100 days. Means with different letters in each sampling day are significantly different (*p* < 0.05, LSD test) considering the effect of IOX concentration. Means without letters did not differ from others on the corresponding sampling day. TPF, triphenyl formazan; IOX, iohexol; AS, activated sludge; LSD, least significant difference.

One of the indicators of AS functioning is the activity of dehydrogenases, which are involved in oxidation and reduction processes both at the level of basic metabolism and in xenobiotic transformation [24]. This study demonstrated considerable fluctuations in dehydrogenase activity depending on incubation time and IOX concentration (Figure 3).

The highest dehydrogenase activity was observed after treatment with 20 mg/L IOX. Two significant decreases in dehydrogenase activity were observed during the study cycle, on days 14 and 56. The reduction observed on day 14 indicates a marked impact of IOX on the microbiological community of AS [25], whereas the decrease on day 56 indicates a temporary disturbance in AS respirometric activity [24]. However, this effect was not directly attributable to IOX, as a similar reduction in dehydrogenase activity was observed in the control, likely due to other components present in the wastewater. Importantly, these changes were not permanent. Notably, IOX at the highest concentration appeared to stimulate dehydrogenase activity. This contrasts with literature reports that describe dehydrogenase activity as a sensitive indicator of environmental pollution [26]. Moreover, it has been reported that diatrizoate, another ICM, negatively affected dehydrogenase activity in AS after 100 days of exposure [19]. This effect may suggest that the structure of iodine contrast compounds significantly affects the functioning of microorganisms in AS, particularly with longer exposure. This may also be related to ICM susceptibility to degradation; diatrizoate is described as less susceptible to physicochemical [27] and biological [28] degradation.

In this study, the increased dehydrogenase activity observed with higher IOX concentrations may be related to IOX transformation. Akao et al. [6] demonstrated that the transformation of this compound by *Chlorella vulgaris* produces aldehyde derivatives (component IDP819) and acid derivatives (component IDP833). These compounds may result from dehydrogenase-mediated oxidation of the hydroxyl groups of IOX, providing a plausible explanation for the higher dehydrogenase activity observed at elevated IOX concentrations.

**Figure 4:**
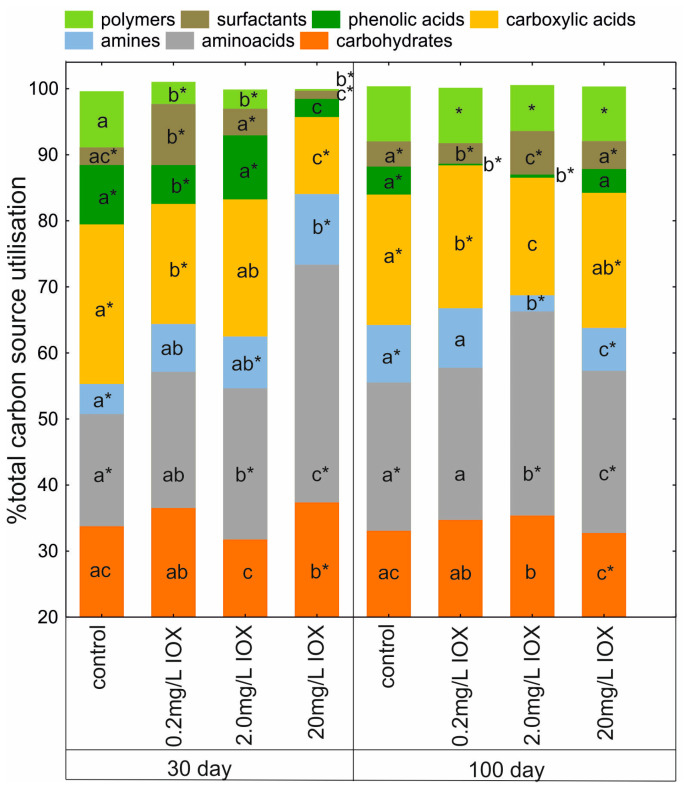
Changes in the utilisation of distinguished groups of carbon sources in control AS and AS treated with IOX (0.2, 2.0, or 20 mg/L) on day 30 and day 100. Means of each carbon source group labelled with different letters are significantly different (*p* < 0.05, LSD test), considering the effect of IOX concentration on each sampling day. Pairs of means with the same IOX concentration marked with an asterisk are significantly different (*p* < 0.05, the independent samples *t*-test), considering the effect of time (day 30 vs. day 100).

Analysis of the metabolic profile of AS in the presence of IOX indicated that a low concentration of 0.2 mg/L did not significantly alter metabolic activity. However, a concentration of 20 mg/L induced significant changes in the metabolic profile of AS (Figure 4).

**Figure 5:**
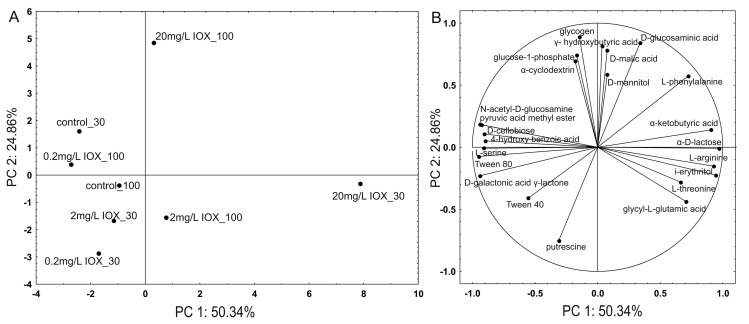
Projection of metabolic profiles of the control AS and AS treated with different IOX concentrations (0.2, 2.0, or 20 mg/L) (**A**) and carbon sources from BiologTM EcoPlatesTM (**B**) along PC1 and PC2. The numbers in captions denote the sampling day. IOX, iohexol; AS, activated sludge; PC, principal component.

Analysis of Gini coefficient values indicated that substrate oxidation was uneven and dependent on IOX concentration. It was also demonstrated that, despite an initial decrease in the number of substances utilized by the AS microbiome after exposure to 20 mg/L of IOX, recovery occurred by day 100, as evidenced by the R coefficient value (Table 1). The most pronounced changes in amino acid metabolism were observed on day 30 after treatment with 20 mg/L IOX (Figure 5).

This likely represents a typical stress response in some bacterial strains and may reflect an adaptation to a toxic environment [29]. Impaired metabolism of phenolic acids was also observed (Figure 5), which may result from the competitive inhibition of enzymes, involved in aromatic compound degradation, by IOX. Due to its large molecular size and electronic structure, IOX may permanently block access of substrates to the catalytic sites of enzymes [30]. The results indicate that AS microorganisms, in the presence of the highest concentration of IOX, regulate amino acid and carbohydrate metabolism and the phosphorus cycle (Figure 5 and Table 1) to maintain energy homeostasis [29]. At lower concentrations, the effect of IOX on the metabolic profile was significantly lower, confirming the low toxicity of IOX toward living organisms [31]. It should be emphasized that significant changes are observed only at high iohexol concentrations, and the mechanism of this toxic effect can only be fully explained after analysis of intermediates.

## 3. Materials and Methods

### 3.1. Sample Collection and Experimental Setup

**Figure 6:**
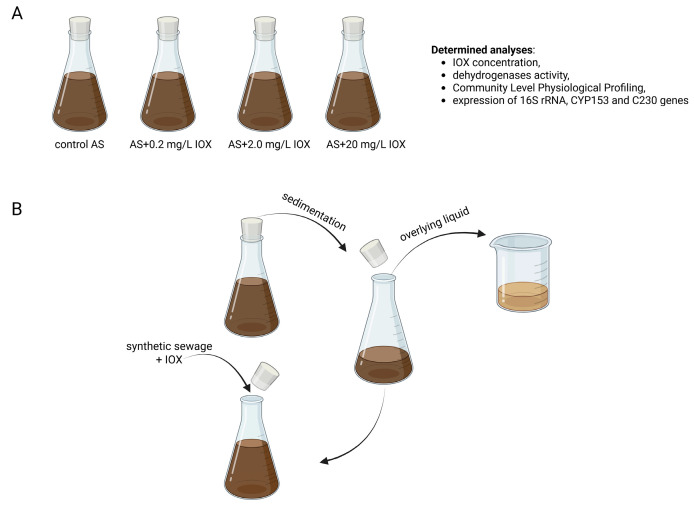
Experimental sets (**A**) and cycle of AS operation (**B**). AS, activated sludge.

The AS was collected from the aeration tank of the wastewater treatment plant Klimzowiec (Katowice, Poland). First, AS was transferred to the 1-L laboratory-scale AS systems in triplicate. Subsequently, AS was contaminated with IOX at concentrations of 0.2, 2.0, and 20 mg/L. AS without IOX addition was treated as the control (Figure 6A). The ‘start’ sample corresponded to the untreated sewage (control AS) at the beginning of the experiment. All sets were maintained at a stable temperature (21 °C) and aerated under shaking conditions (130 rpm) for 100 days. At 7-day intervals, AS was sedimented for 30 min, the overlying liquid was removed, and AS was supplemented with fresh synthetic sewage (composition according to [32] and the appropriate amount of IOX to achieve the intended initial concentrations (Figure 6B).

### 3.2. Sewage Physico-Chemical Analysis

Physico-chemical parameters of sewage (pH, chemical oxygen demand (COD), biological oxygen demand (BOD), sludge volume index (SVI), dry residue (DR), total suspended solids (TSS), total organic carbon (TOC), PO_4_^−^, total nitrogen, ammonium nitrogen (N-NH_4_^+^), nitrite nitrogen (N-NO_2_^−^), nitrate nitrogen (N-NO_3_^−^), chlorides and sulfates) were determined according to the standards at the accredited chemical laboratory (Eurofins OBiKŚ Poland Sp. z o.o., Katowice, Poland). All methods are listed in our previous study [19].

### 3.3. Measuring Dehydrogenase Activity

The activity of AS microorganisms was determined by quantifying dehydrogenase activity (EC 1.1.1) based on the reduction of 2,3,5-triphenyltetrazolium chloride (TTC) to insoluble, red triphenyl formazan (TPF), following the method of Miksch [33].

### 3.4. Determination of IOX Concentration

The concentration of IOX in AS was monitored using reversed-phase high-performance liquid chromatography (RP-HPLC) (Nexera LC-40, Shimadzu, Kyoto, Japan) equipped with a ReproSil-Pur Basic C18 column (150 × 4.6 mm, 5 µm; Dr Maisch HPLC GmbH, Ammerbuch, Germany) and a photodiode array detector. For IOX determination, the mobile phase consisted of acetonitrile and 1% acetic acid (5:95 *v*/*v*), and the sample volume was 10 µL. Separation was conducted under isocratic conditions at a flow rate of 1 mL/min, with a detection wavelength of 245 nm. The IOX concentration was calculated from the calibration curve described by the equation y = 15372x (R^2^ = 0.999), which was linear in the concentration range of 0–100 mg/L. LOD/LOQ value was 3.03. Since the binding of iohexol to the biomass was negligible (<5%), the result obtained in chromatographic measurements was taken as 100% of the compound, assuming a measurement uncertainty of 5% of the result. Samples were collected once a week before AS sedimentation and centrifuged at 14,000 rpm for 25 min prior to HPLC analysis.

### 3.5. Community-Level Physiological Profiling

The community-level physiological profiles in the AS samples were assessed using 96-well Biolog^TM^ EcoPlates^TM^ (Biolog Inc., Hayward, CA, USA) and a Spark^TM^ Multimode Microplate Reader (Tecan Group Ltd., Männedorf, Switzerland). Samples (10 mL) were collected from each AS system at the start of the experiment and after 30 and 100 days. They were subsequently homogenised for 10 s at 11,000× *g* using an Ultra-Turrax T-25 digital homogeniser (IKA Works, Guangzhou, China) and diluted 100-fold with sterile 0.9% NaCl. Subsequently, 120 µL of each suspension was used to inoculate each well of the EcoPlates. The plates were incubated at 21 °C in the dark for five days. Absorbance in each well was measured at 590 nm at the beginning and at equal time intervals. Functional diversity indices were calculated following the methods of Nowak and Mrozik [34], and the Gini coefficient (G) was calculated according to Al-Mutairi [35]. The carbon sources utilised in EcoPlates were grouped into six guilds [34].

### 3.6. RNA Extraction and Reverse Transcription Quantitative Polymerase Chain Reaction (RT-qPCR) Analysis

RNA was extracted from AS samples (n = 3 per treatment) using the RNeasy PowerSoil Total RNA Kit (Qiagen, Germantown, MD, USA) according to the manufacturer’s instructions. RNA concentration and purity were measured with an Implen NanoPhotometer spectrophotometer (Implen GmbH, Munich, Germany). Total RNA was treated with RQ1 RNase-free DNase (Promega, Madison, WI, USA; 1 U/μg RNA, 30 min at 37 °C). The efficiency of the DNase treatment was validated by confirming that DNase-treated RNA showed no amplification in no-RT controls (Cq > 40), whereas untreated RNA produced a clear amplification signal. cDNA synthesis was performed using the RevertAid First Strand cDNA Synthesis Kit (Thermo Scientific™, Waltham, MA, USA). The purified RNA samples were stored at −80 °C, and the resulting cDNA was kept at −20 °C.

Quantification of the expression levels of the *16S rRNA* gene and genes encoding cytochrome P450-type alkane hydroxylase (*CYP153*) and catechol 2,3-dioxygenase (*C23O*) was performed using the RT-qPCR method with specific primer sets (Table 3) [36,37,38], using a LightCycler^®^ 96 Real-Time PCR System (Roche Diagnostics, Florham Park, NJ, USA).

Briefly, the 10 µL reaction mixture contained 2 µL of cDNA, 1 µL of the primer pair mixture (10 µM), and 5 µL of 2 × Master Mix (FastStart Essential DNA Green Master) (Roche Diagnostics, Florham Park, NJ, USA). No-template controls were included for every primer set to check reagent contamination and primer-dimer formation. The amplification protocol was as follows: initial denaturation for 10 min at 95 °C, followed by 30 cycles of 10 s at 95 °C, 20 s at the specific annealing temperature (given in Table 3), and 30 s at 72 °C. Fluorescence data were collected at 81 °C at the end of each extension step to prevent the detection of primer dimers. To perform the melting curve analysis of the products, the temperature was raised from 65 to 95 °C. Standard curves were generated from five-point, tenfold serial dilutions of the pJET1.2/blunt plasmid (Thermo Scientific™, Waltham, MA, USA) carrying each target gene. Each dilution was run in triplicate. The slopes, R^2^ values, and amplification efficiencies were calculated from the linear regression of Cq versus log_10_ template copy number, using the equation E = (10^(−1/slope)^ − 1) × 100%. All primer sets showed efficiencies ranging from 90% to 110% and R^2^ values ≥ 0.98.

### 3.7. Data Analysis

Statistical analyses were conducted using STATISTICA 13.1 PL (TIBCO Software Inc., Palo Alto, CA, USA). One-way analysis of variance (ANOVA) was applied to assess differences between control and treated AS samples collected on the same measurement day, with statistical significance set at *p* < 0.05. To evaluate the impact of exposure duration (30 vs. 100 days), data from AS samples treated with the same concentration of IOX were compared using an independent samples *t*-test (*p* < 0.05). Additionally, principal component analysis was employed to identify differences in microbial metabolic profiles among the experimental setups.

The limit of Determination (LOD) and the Limit of Quantitation (LOQ) were calculated according to equations: LOD = 3.3·σ/S and LOQ = 10·σ/S, respectively, where σ—the standard deviation of the response and S—the slope of the calibration curve [39].

## 4. Conclusions

In summary, the study results demonstrate that the AS microbiome can transform IOX; however, the efficiency of this process declines over time. Despite the reduced biotransformation capacity, the long-term exposure to higher IOX concentrations led to an increase in functional diversity and microbial activity, as confirmed by the Shannon index and dehydrogenase activity. These findings may indicate that IOX does not act as a selective agent targeted microorganisms, likely due to its low toxicity. Overall, IOX did not significantly impact AS functioning or structure, with only the highest concentration (20 mg/L) producing minor effects on sludge biochemistry. Due to the heterogeneity of activated sludge, the study focused on the basic metabolic capabilities of activated sludge, including carbon source metabolism, metabolic activity, and reducing activity, to ensure that the results could be directly applied to other systems. However, full validation of the obtained data requires further research, including metagenomic studies. The answer to whether activated sludge functioning in the presence of iohexol represents a typical response of AS microbiomes will allow for the prediction of the effects of iodinated contrast agents in wastewater treatment plants. The answer to this question is all the more important because the widespread use of these agents will, in the future, increase their concentration in wastewater and water.

## Figures and Tables

**Table 1:** Functional capacity indices for the control AS and AS contaminated with IOX (0.2, 2.0, and 20 mg/L) at the beginning, day 30, and day 100 of exposure. The numbers in the captions denote the sampling day. In each column, means with different letters are significantly different (*p* < 0.05, LSD test), considering the effect of IOX concentrations separately. IOX, iohexol; AS, activated sludge; LSD, least significant difference.

	G	H′	R	AWCD	N-Use, %	P-Use, %
start	0.54 ± 0.02	1.27 ± 0.02	27.5 ± 1.6	1.11 ± 0.14	35.8 ± 4.2	3.76 ± 0.42
control_30	0.60 ± 0.07 ^a^	1.25 ± 0.02 ^a^	24.0 ± 2.2 ^a^	0.93 ± 0.07 ^a^	28.8 ± 0.5 ^a^	7.86 ± 0.00 ^a^
0.2 IOX_30	0.71 ± 0.11 ^b^	1.27 ± 0.01 ^ab^	21.5 ± 4.9 ^ab^	0.84 ± 0.21 ^a^	33.2 ± 4.7 ^c^	0.09 ± 0.05 ^b^
2.0 IOX_30	0.59 ± 0.09 ^a^	1.29 ± 0.03 ^b^	23.5 ± 4.9 ^ab^	0.84 ± 0.22 ^a^	35.9 ± 3.5 ^bc^	0.98 ± 0.09 ^c^
20 IOX_30	0.87 ± 0.09 ^c^	1.05 ± 0.01 ^d^	20.0 ± 4.4 ^b^	0.24 ± 0.08 ^b^	38.2 ± 5.6 ^b^	1.89 ± 0.00 ^d^
control_100	0.54 ± 0.09 ^a^	1.34 ± 0.01 ^a^	25.5 ± 2.7 ^ac^	0.80 ± 0.19 ^a^	34.7 ± 4.1 ^a^	7.01 ± 2.60 ^a^
0.2 IOX_100	0.64 ± 0.02 ^b^	1.24 ± 0.01 ^b^	19.5 ± 1.6 ^b^	0.75 ± 0.02 ^a^	33.8 ± 2.7 ^a^	0.00 ± 0.00 ^b^
2.0 IOX_100	0.45 ± 0.01 ^c^	1.33 ± 0.01 ^a^	23.5 ± 1.6 ^a^	1.34 ± 0.05 ^b^	40.3 ± 3.7 ^b^	0.00 ± 0.00 ^b^
20 IOX_100	0.46 ± 0.10 ^ac^	1.37 ± 0.00 ^c^	26.0 ± 0.0 ^c^	1.04 ± 0.21 ^c^	33.5 ± 2.4 ^a^	10.73 ± 0.50 ^c^

**Table 2:** Physico-chemical characteristics of sewage at the beginning and at the end (day 100) of the experiment. All parameters, except pH and SVI (mL/g) are expressed in mg/L. The sample start is untreated sewage at the beginning of the experiment; the control is untreated sewage after 100 days of incubation; 0.2 mg/L IOX, 2 mg/L IOX, and 20 mg/L IOX are sewage samples exposed to the respective concentrations of IOX for 100 days. IOX, iohexol.

Parameter	Start	Control	0.2 mg/L IOX	2 mg/L IOX	20 mg/L IOX
pH	6.7 ± 0.2	7.4 ± 0.2	7.0 ± 0.2	7.4 ± 0.2	7.2 ± 0.2
COD	2880 ± 432	988 ± 148	1570 ± 236	1300 ± 195	1500 ± 225
BOD	570 ± 114	150 ± 30	43 ± 9	120 ± 24	230 ± 46
SVI	34.3 ± 6.8	76.9 ± 15.2	68.3 ± 13.6	89.7 ± 17.4	90.2 ± 18.0
TSS	>4000 ± 40	1200 ± 117	1600 ± 161	1600 ± 156	1300 ± 133
TOC	825 ± 165	431 ± 86	499 ± 100	307 ± 61	457 ± 91
DR	5030 ± 503	1930 ± 193	2430 ± 243	3150 ± 315	2000 ± 200
PO_4_^−^	129 ± 19	47.5 ± 7.1	50.2 ± 7.5	44.2 ± 6.6	42.6 ± 6.4
Total N	139 ± 1	78 ± 12	100 ± 15	100 ± 15	74 ± 11
N-NH_4_^+^	23 ± 2	<0.1 ± 0.02	0.22 ± 0.06	0.17 ± 0.04	0.21 ± 0.01
N-NO_2_^−^	0.23 ± 0.02	0.04 ± 0.01	0.07 ± 0.03	0.02 ± 0.01	0.06 ± 0.02
N-NO_3_^−^	4.0 ± 0.6	29 ± 4	41 ± 6	34 ± 5	27 ± 4
Chlorides	140 ± 14	150 ± 15	180 ± 18	170 ± 17	150 ± 15
Sulfates	96 ± 10	60 ± 6	61 ± 6	54 ± 5	54 ± 5

**Table 3:** Primers used for PCR amplification.

Primer	Sequence (5′-3′)	Gene	Annealing (°C)	Reference
pE pF•	AAACTCAAAGGAATTGACGG ACGAGCTGACGACAGCCATG	*16S rRNA*	57	[36]
P450fw1 P450rv3	GTSGGCGGCAACGACACSAC GCASCGGTGGATGCCGAAGCCRA	*CYP153*	58	[38]
C23OF C23OR	AAGAGGCATGGGGGCGCACCGGTTCGA TCACCAGCAAACACCTCGTTGCGGTTGCC	*C23O*	57	[37]

## Data Availability

The data presented in this study are available on request from the corresponding author.
